# Biological Evaluation of Flexible Polyurethane/Poly l-Lactic Acid Composite Scaffold as a Potential Filler for Bone Regeneration

**DOI:** 10.3390/ma10091042

**Published:** 2017-09-13

**Authors:** Yuk Fai Lui, Wing Yuk Ip

**Affiliations:** Department of Orthopaedics and Traumatology, The University of Hong Kong, Hong Kong, China

**Keywords:** biomaterial, bone graft substitute, bone filler

## Abstract

Degradable bone graft substitute for large-volume bone defects is a continuously developing field in orthopedics. With the advance in biomaterial in past decades, a wide range of new materials has been investigated for their potential in this application. When compared to common biopolymers within the field such as PLA or PCL, elastomers such as polyurethane offer some unique advantages in terms of flexibility. In cases of bone defect treatments, a flexible soft filler can help to establish an intimate contact with surrounding bones to provide a stable bone-material interface for cell proliferation and ingrowth of tissue. In this study, a porous filler based on segmented polyurethane incorporated with poly l-lactic acid was synthesized by a phase inverse salt leaching method. The filler was put through in vitro and in vivo tests to evaluate its potential in acting as a bone graft substitute for critical-sized bone defects. In vitro results indicated there was a major improvement in biological response, including cell attachment, proliferation and alkaline phosphatase expression for osteoblast-like cells when seeded on the composite material compared to unmodified polyurethane. In vivo evaluation on a critical-sized defect model of New Zealand White (NZW) rabbit indicated there was bone ingrowth along the defect area with the introduction of the new filler. A tight interface formed between bone and filler, with osteogenic cells proliferating on the surface. The result suggested polyurethane/poly l-lactic acid composite is a material with the potential to act as a bone graft substitute for orthopedics application.

## 1. Introduction

In clinical terms, a critical-sized bone defect is defined as, “The smallest size tissue defect that will not completely heal over a natural lifetime” [1]. Natural healing of these defects is usually limited by their large involved volume, which prohibits cell proliferation and signal transmission [2]. The formation of these critical defects can be caused by external factors such as a physical fracture, or a pathological disorder such as trauma or malignancy [3]. Direct consequences include pain and anatomical deformation and, in some cases, significant long-term morbidity. In most of the clinical cases, trauma is considered as the major cause of large-volume permanent bone loss, which leads to non-union and structural failure in the skeletal system of the patient [4]. How to promote healing and osteogenesis inside these defects has been a common topic within clinical sectors for the past 40 years. Traditional vascularized autogenic bone grafting has served as the gold standard treatment for promoting regeneration in general bone defects. A limited supply and donor-site morbidity of autogenic bone grafts, however, have created obstacles in many clinical cases, especially when large-volume defects are involved [5,6,7,8,9]. For the past decade, with the advance in tissue regeneration therapies, many novel techniques had been developed for promoting healing in critical-sized bone defects [8,10,11,12,13,14,15,16,17,18,19,20,21,22]. Examples of new treatments under development include intramedullary lengthening devices, bioactive membranes, osteogenic proteins and tissue engineering [8,22,23,24,25,26,27,28,29,30].

One promising new technique for promoting healing in these large-volume defects is bone-graft substitutes. A bone-graft substitute, in general, is an artificially introduced filler that provides an anchoring point for cell proliferation and temporary structural support for tissue ingrowth, which in the meantime can be integrated into, or completely replaced by, newly formed tissue after the healing process is complete [31,32]. Traditionally, ceramic is the most favorable material as a bone-graft substitute because of its great osteoconductivity and osteoinductivity. Various kinds of hydroxyapatite and TCP-based fillers are undergoing investigation and clinical trial for their potential in facilitating bone healing [8,9,32,33,34]. On the other hand, developments in biomaterial have opened up new material choices within this field. Polymers are a new material family that has appeared on the list of artificial bone-graft substitutes [24,35,36,37]. When compared to traditional high-strength fillers such as ceramics and metals, polymers offer advantages in biodegradability and flexibility [35,38]. A great advantage of polymer filler over metal and ceramics is that it can be used to fill a larger variety of defects of different sizes and shapes thanks to its ease of fabrication. Many different types of polymer have undergone investigation for their potential in regeneration medicine applied to orthopedics [12,35]. Some common candidates such as poly-l-lactic acid and poly-l-glycolic acid have shown high compatibility with living tissue and can induce a significant effect on osteoconduction [39,40,41,42,43].

In our study, a new polyurethane composite is investigated for its potential as a filler in critical-sized bone defects. Polyurethane has a long history within in vivo application, such as artificial heart valves [44,45,46,47]. It is a non-toxic material that induces little or no negative effects in a living body [48,49,50,51]. However, compared to the traditional polymers mentioned above, polyurethane is a less popular material for regeneration medicine. Despite possessing unique physical properties favorable for some clinical applications [44,52,53,54,55], a few setbacks have concealed the full potential of polyurethane as a degradable filler. Polyurethane is a degradable polymer in vivo but is subject to a slow degradable rate that can last for a long time after implantation [56,57]. The hydrophobicity and bio-inertness of polyurethane also results in a low cell adhesion coefficient when compared to other biopolymers, which can affect the proliferation of cells [58].

Nevertheless, polyurethane has a soft nature that is unique among synthetic biopolymers. A polyurethane scaffold can be deformed under external stress without losing integrity and is able to restore its dimensions after deformation. This is not only convenient during implantation surgery, but also allows better localization inside the desired area during the healing process, especially in an area that involves active movement and constantly changing external stress. The soft texture also enables the material to tightly fill the defects and establish a stable interface with surrounding tissue, which is beneficial for new tissue formation [59]. The integrity of polyurethane when subjected to external stress also offers a structurally stable environment for tissue growth. These unique properties offer polyurethane an advantage over the majority of stiff and brittle biopolymers when acting as a tissue filler in large-volume defects [50,59].

In this study, the potential of polyurethane-based material as a bone filler for osteogenesis in critical-sized defects is evaluated. To counter the shortcomings of polyurethane, poly l-lactic acid was incorporated to improve tissue responses including cell compatibility and in vivo degradation rate [60]. It was hypothesized that the improvement in biological response could lead to improvement in osteoconductivity and osteoinductivity of the polyurethane scaffold. In vitro and in vivo investigation were conducted to evaluate the potential of the new bone filler in orthopedic applications.

## 2. Material and Methods

### 2.1. Fabrication of Polyurethane/Poly l-Lactic Acid Filler

S Segmented polyurethane [61] with average molecular weight 1 × 105 was dissolved in 99% N,N-Dimethylformamide (Sigma Aldrich, St. Louis, MO, USA, cat no. #D158550-4L) at 80 °C with continuous stirring for 24 h to form a polyurethane solution of 9.5% w/v. Undissolved raw polymer was removed by suction filtration with a glass filter. An 8% w/v poly-l-lactic solution was prepared by dissolving poly-l-lactic granules (Goodfellow Cambridge, Huntingdon, UK, LS399664) into chloroform (Merck, Kenilworth, NJ, USA, 1.02455.2500) under vertex. During stirring, the poly-l-lactic acid solution was added to the polyurethane solution drop by drop. The solution was removed from the heat source immediately. Sodium chloride in a volume ratio of about 1:1.5 was added slowly to the composite solution with extensive stirring until the first sight of polymer displacement. The mixture was allowed to solidify and dry for 7–14 days and was then transferred to a 60 °C oven until all the solvent had evaporated. After solidification, the mixture was under dialysis with deionized water for 48–72 h until all the sodium chloride particles were dissolved and removed from the remaining scaffold.

Five different PL/PU composites with different composition ratios were investigated. The composition ratio is summed up in Table 1.

### 2.2. Culture of Osteoblast and Cell Seeding

7F2 mouse osteoblast obtained from ATCC (ATCC CRL-12557, Manassas, VA, USA) was subcultured and expanded under general protocol. Prior to cell seeding, the culture medium was discarded and the monolayer rinsed with 1× sterilized phosphate buffer solution. 0.25% w/v trypsin was added to the monolayer and culture at 37 °C for 5 min until the cell layer was dispersed. Dispersed cells were diluted to 1 M cell/mL before cell seeding.

Scaffolds were cut into circles 11 mm in diameter and 2 mm in thickness using a sharp blade. The samples were then packed with double packing and sterilized by autoclave before cell seeding. Prior to cell seeding, sterilized samples were immersed in 0.5 mL culture medium in a 48-well culture plate for 72 h to allow perfusion of the medium. All samples were anchored by press fitting inside wells. 5 × 104 cells per well were seeded on the samples with Dulbecco’s Modified Eagle Medium (high glucose) supplemented with 10% fetal bovine serum as the growth medium. The culture medium was renewed every three days.

### 2.3. Cell Viability Assay

MTT cell proliferation assay was performed directly inside the 48-well culture plates. Before the test, 5 mg of MTT 3-(4.5-dimethylthiazol-2-yl)-2,5-diphenyltetrazolium bromide was dissolved in 1 mL of sterilized phosphate buffer solution to form a 12 mM MTT solution. 1 gm of Sodium dodecyl sulfate was dissolved in 10 mL of 0.01 M HCl to form SDS solution. Old medium was removed from the culture plate by suction pump. Samples were washed with 1× phosphate buffer solution to remove residue medium and 1 mL of flesh medium was added. 100 μL of 12 mM MTT solution was added to each well. The culture plate was covered in aluminum foil and incubated in darkness at 37 °C for 4 h. After incubation, 1 mL of SDS-HCl solution was added to each well to release the pigment. The plate was incubated at 37 °C for 18 h inside a humidified incubator (Model 371, Thermo Fisher Scientific, Waltham, MA, USA). After incubation, the content of each well was mixed using a pipette. 200 uL content from each well was then transferred to a 96-well transparent culture plate. Absorbance at 570 nm was recorded by Thermo Varioskan microplate reader (Varioskan LUX Multimode Microplate Reader, Thermo Fisher Scientific, Waltham, MA, USA).

### 2.4. Alkaline Phosphatase Assay

Standard solution was prepared from 0.1 mg/mL alkaline phosphate control enzyme (Sigma Aldrich cat no C9361, St. Louis, MO, USA) starting with a 1:100 dilution. A standard curve was prepared from the diluted standard solution according to the following concentrations: 100%, 50%, 25%, 12.5%, and 6.25%. A substrate solution was prepared by dissolving 1 mg of 4-methylumbelliferyl phosphate disodium (Sigma Aldrich cat no M8168, St. Louis, MO, USA) in 330 μL of deionized water. Samples were washed gently with 1× phosphate buffer solution. Cell lysate was prepared by adding 1 mL of cell lysate buffer to each sample. The samples with cell lysate buffer were then placed in a 4 °C refrigerator for 30 min. After the cytolysis, the cell lysate was cooled to room temperature. 20 μL of each sample was transferred to a black 96-well culture plate. To each well, 20 μL of dilution buffer (Sigma Aldrich cat no B6433, St. Louis, MO, USA) and 160 μL of fluorescent assay buffer (Sigma Aldrich cat no B6558, St. Louis, MO, USA) were added. Before plate reading, 1 μL of the 10 nM substrate was adder to each well and mixed. The plate was read under Thermo Varioskan microplate reader at 360 nm excitation and 440 nm emission.

### 2.5. Energy Dispersive X-ray Spectroscopy

Collected samples were washed with 1× sterilized PBS to remove all residue medium. Samples were fixed with 10% for 30 min. After fixation, all samples were dehydrated according to the following protocol; 70% ethanol (20 min, two changes), 80% ethanol (20 min, two changes), 90% ethanol (30 min, three changes) and 100% ethanol (30 min, three changes). The samples were dried and stored inside a desiccator to remove all moisture on the surface before the imaging process. 1 μm-thick gold particle was coated on the surface of the samples for better electron conductivity.

### 2.6. In Vivo Evaluation on Artificial Created Critical Sized Defect

**Figure 1:**
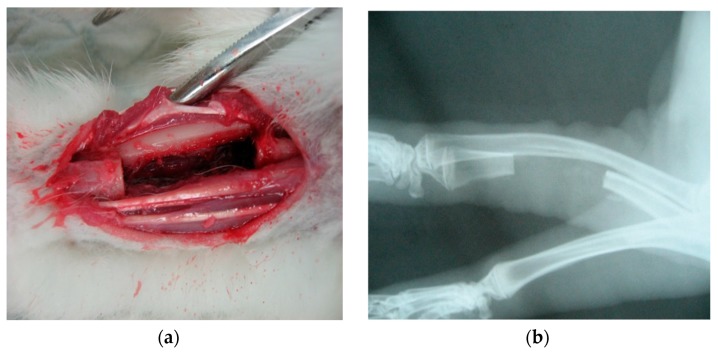
Critical-sized bone defect created on ulna of NZW rabbits. (**a**) Surgical sight; (**b**) Under X-ray imaging.

A NZW rabbit at 9-week-old and weighing about 2.5 kg was chosen as the subject for in vivo evaluation. An artificial defect of 20 mm was created on left radius of the animal. (Figure 1) The operation began by giving the rabbit a preoperative cocktail of antibiotics and sedation, which included 15 mg/kg subcutaneous cephalexin, intramuscular ketamine (35 mg/kg), Xylazine (5 mg/kg) and acepromzine (1 mg/kg). Before the surgical operation, procedures such as hair shaving and skin disinfection were carried out on the surgical site. An incision was created above the distal radius. Muscle was separated to expose the bone. A segmental defect of 20 mm was created by surgical saw under constant cooling with saline. After removal of a radius segment, the polyurethane composite fillers were implanted into the defect by press fitting without any further external or internal support. After implantation, the wound was closed by simple suture and the forearm was fixed by bandage. The animal was allowed to recover under normal nursing care with a post-operation treatment of 0.1 mg/kg buprenorphine and 15 mg/kg enrofloxacin for 5 days.

### 2.7. Tissue Processing after Sacrifice of Animal

At the respective time, the rabbit was sacrificed with an overdose pentobarbital. A large section of radius including the defect site was harvested and fixed in 10% formalin buffer solution for 1 week before dehydrate in ethanol (70%, 95%, 100%). After dehydration, the sample was infiltrated with xylene for 48–96 h and then embedded with MMA. Embedded samples were cut into sections by an embedded diamond cutting band system complete with precision parallel control contact point (EXAKT 300CP, Oklahoma City, OK, USA) to a thickness of about 0.3 mm. The sections were attached on transparent slices by methyl methacrylate precision adhesive (EXAKT 7210 VLC, Oklahoma City, OK, USA). The sections were polished by micro grinding to a final thickness of about 0.07 mm. Polished sections were dried for histological staining.

## 3. Results

### 3.1. Osteogenic Cellular Response on Polyurethane Composite

7F2 osteoblast was employed for evaluation of the osteogenic cellular response on the polyurethane/poly l-lactic acid composite filler through a 21-day culture test. Evaluation was based on cell attachment, proliferation and osteogenesis markers. Culture was performed in 48-well culture plate following the standard protocol listed before. Quantitative analysis was performed on days 3, 7, 14 and 21 after cell seeding.

#### 3.1.1. Cell Attachment

**Figure 2:**
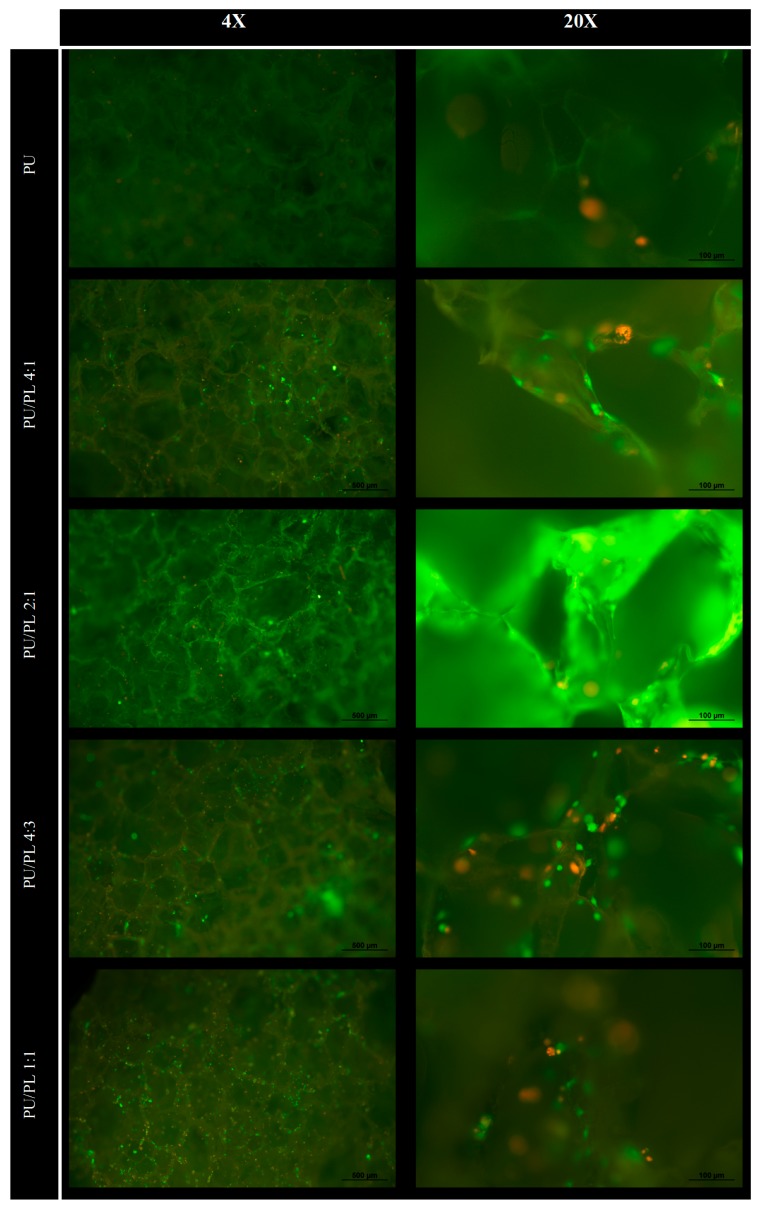
7F2 osteoblast cultured on polyurethane composite fillers with different composition under LIVE/DEAD stain, 3 days after cell seeding.

**Figure 3:**
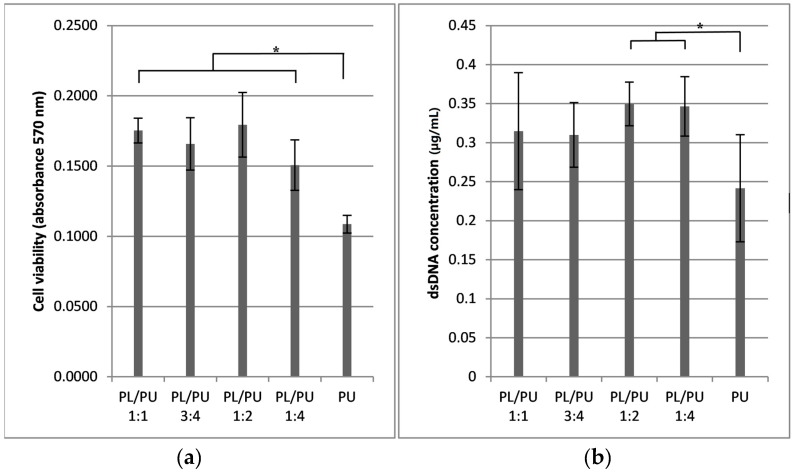
(**a**) Cell viability and; (**b**) dsDNA level in 7F2 culture on polyurethane composite 3 days after seeding, measured by MTT cell proliferation assay and pico-green assay respectively (*n* = 5) (* *p* > 0.05).

The presence of living cells on the investigated samples could be confirmed by fluorescent LIVE/DEAD staining. It is difficult to make an accurate quantitative judgment due to the porous system of the filler, which made wide angle focusing impossible, but there was an obvious improvement in cell seeding efficiency in all polyurethane composition samples when compared to unmodified polyurethane under microscopy. The difference in the number of cells could have been more than 1000 times in certain samples. (Figure 2) Cell distribution was not even, but scattered randomly all over the sample, with most of the living cells located on the horizontal surface of the pores. This finding could be further confirmed by a higher cell viability level under MTT assay (Figure 3). Recorded absorbance in culture on the PL/PU 1:4 composite was about 38% higher than culture on unmodified polyurethane. The dsDNA quantity in culture at day 3 of the experiment was also coherent with the above data, where a 43% increase could be detected when compared to the culture on the unmodified polyurethane.

#### 3.1.2. Cell Activities and Proliferation

**Figure 4:**
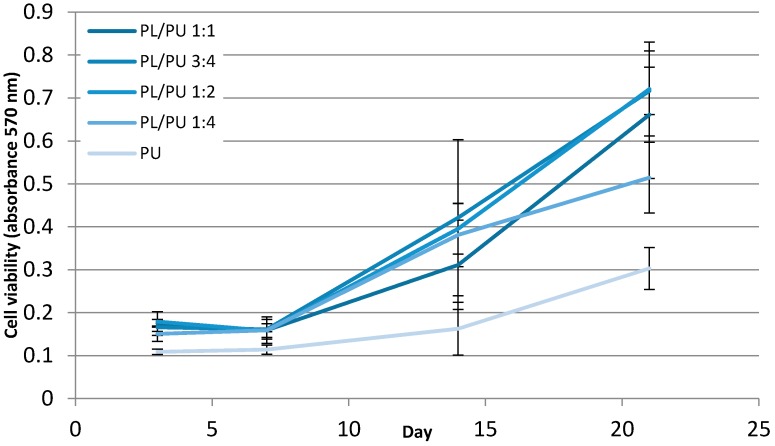
Cell viability level throughout a 21-day culture period of 7F2 cells on polyurethane composites, measured with MTT cell proliferation assay (*n* = 5).

A higher cell activity level could be detected in culture on all polyurethane composite samples. MTT cell viability assay indicated a minimal 38% higher cell activity level for culture on composite fillers (for further information, activity level equalled 30% of positive control) when compared to unmodified polyurethane (Figure 4). There was no or only a slight increase in cell activity level detected between days 3 and 7. Cell activity level increased sharply after day 7 in all experimental groups. The rate of increase of cell activity was higher on modified polyurethane composite groups. Cell activity of all the cultures of composite filler groups had at least doubled to those on unmodified polyurethane fillers by day 14. During the 21-day experiment, a continuous increase in cell activity level could be detected in all experimental groups. The activity level of the culture of composite fillers remained significantly higher than those of unmodified polyurethane.

#### 3.1.3. Osteogenic Response

**Figure 5:**
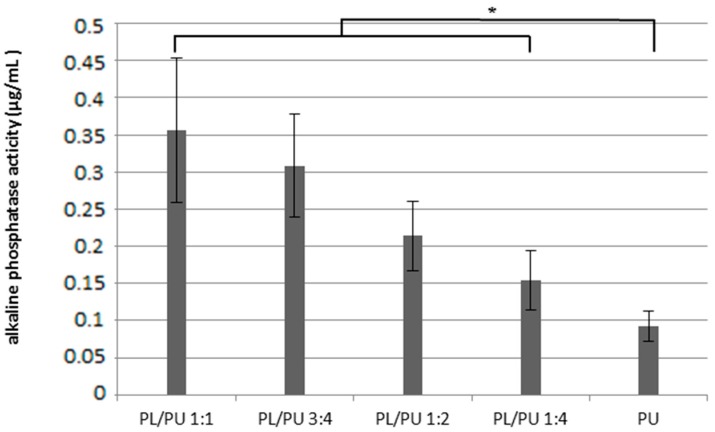
Alkaline phosphatase activity expressed in 7F2 culture on polyurethane composite 3 days after seeding (*n* = 5) (* *p* > 0.05).

**Figure 6:**
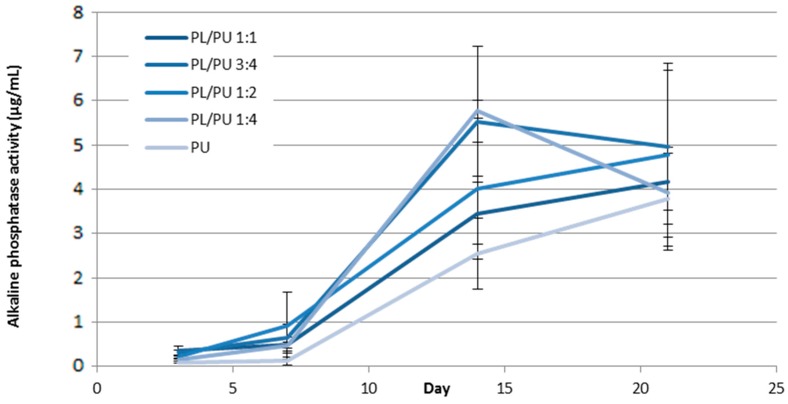
Alkaline phosphatase activity throughout a 21-day culture period expressed in 7F2 cells on polyurethane composites (*n* = 5).

Overall, a higher alkaline phosphatase activity was detected in culture on polyurethane composite fillers than on unmodified polyurethane. At day 3 of the experiment, at least 2.3 times higher alkaline phosphatase activity was recorded in collected cell lysate on polyurethane composite than on unmodified polyurethane fillers (Figure 5). A lower activity was detected in culture on samples with lower poly l-lactic acid content. The highest activity, which was about 48% of the activity level of the positive control, was detected in culture on sample PL/PU 1:1. Alkaline phosphatase activity of culture was in general higher in composite groups throughout the in vitro culture period. At day 14 the activity level for culture on the composite fillers was an at least 35% higher (Figure 6).

**Figure 7:**
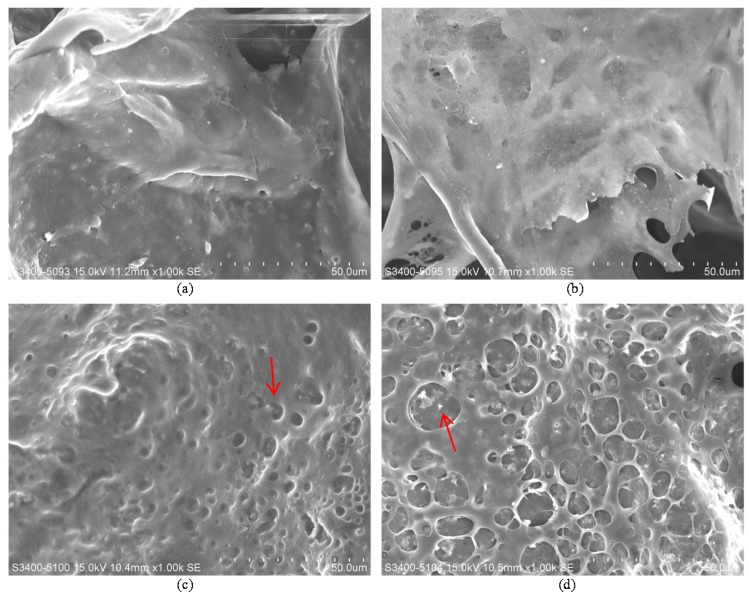
Surface morphology of polyurethane composites after 7F2 culture for 21 days: (**a**) PL/PU 1:4; (**b**) PL/PU 1:2; (**c**) PL/PU 3:4; (**d**) PL/PU 1:1. There was generally an increase in extracellular matrix thickness with the increase in poly l-lactic acid concentration.

**Figure 8:**
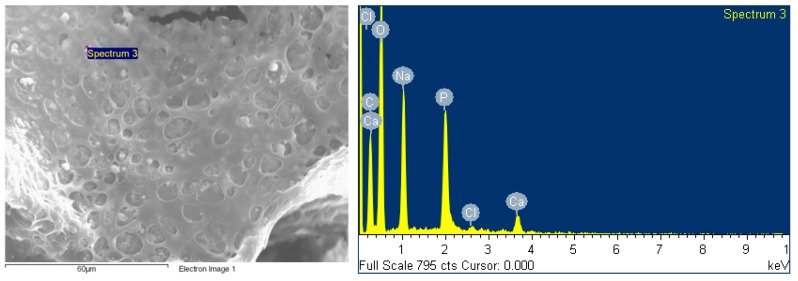
Energy dispersive X-ray spectroscopy result obtained on 7F2 osteoblast-seeded polyurethane composite filler.

Under a scanning electron microscope, extensive formation of an extracellular matrix could be found on the surface, which appeared in the form of a porous membrane. Compared with unmodified polyurethane fillers, the extracellular matrix deposition was more active and covered nearly the whole surface of the sample (Figure 7). Calcium deposition could be confirmed using energy-dispersive X-ray spectroscopy early in day 3, but was much more abundant by day 21 of the culture on all samples (Figure 8). Most of the calcium appeared as calcium oxide-based crystals located inside the pores of an extensive extracellular matrix covering the material surface.

#### 3.1.4. Establishment of a Critical-Sized Bone Defect Model in NZW Rabbit

**Figure 9:**
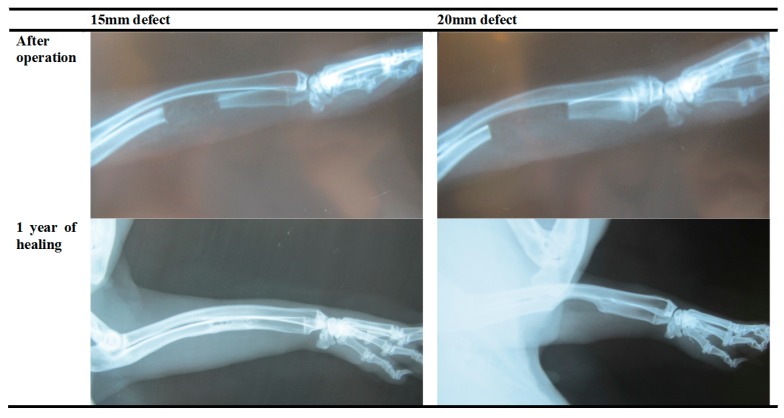
Segmental defect created on NZW rabbit after 1 year of healing.

**Figure 10:**
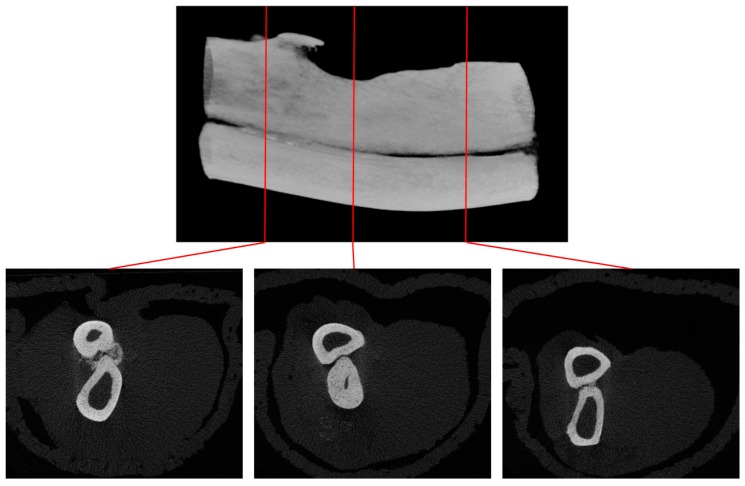
1.5 mm defect after 1 year of healing time micro CT-scanning. There is almost complete restoration of defect volume with the formation of a separate ulna in this particular case. There exist other animal subjects where there is fusion in the ulna and radius.

**Figure 11:**
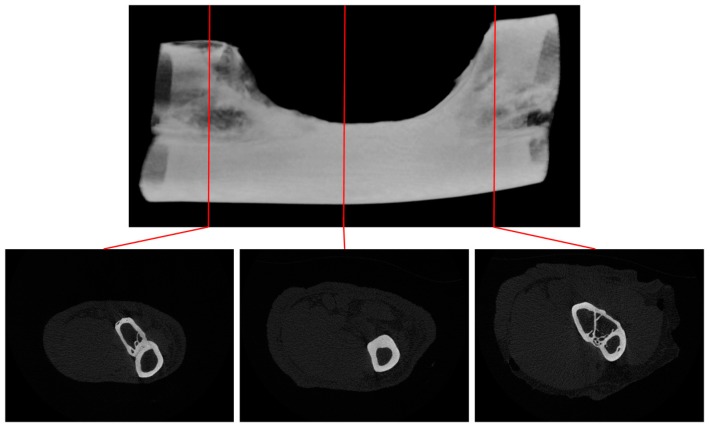
20 mm defect after 1 year of healing time micro CT-scanning. There is no restoration in most of the defect volume. Deformation in the cortex is revealed in the segmental end of ulna, where there is a fusion of the radius and ulna. Inside the defect there is a change in anatomy of the radius, resulting in a thicker cortex.

Prior to the main study, a critical bone defect model was established. From the results obtained in the prior study, a critical-sized defect can be achieved by removing about 20 mm of bone from the ulna. There is almost complete recovery for any defect of less than 1.5 mm after 1 year of healing even without any artificial influences, contrary to the model widely employed in various literatures [62,63,64,65]. (Figure 9) This finding can also be confirmed under CT-scanning (Figure 10 and Figure 11).

#### 3.1.5. In Vivo Evaluation on Artificial Created Critical-Sized Defect

**Figure 12:**
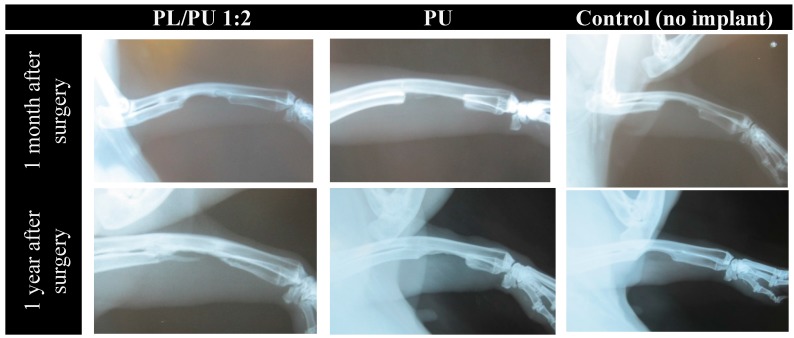
Healing process of critical-sized bone defect in NZW rabbit model with different implants.

Only PL/PU2:1 was chosen for further animal study because the composition causes little alternation in physical properties while having a significant positive effect on biological response. One-year investigation of critical-sized bone defects facilitated with composite polyurethane fillers was conducted on NZW rabbits. Continuous inspection of the healing process using X-ray images indicated that there was no, or only slight, dislocation of bones during the healing process. The broken ulna remained in its original orientation. The early stage of healing shared many similarities with healing in typical critical-sized defects. One month after the surgical operation, new calcification tissue could be observed in an area in between the radius and ulna near both ends of the defect. The calcification took place around the original cortical bone, which forms a callus around the defected ulna. Two months after the surgical operation, there were traces of remodeling of the ulna and radius in both defect ends, which eventually caused fusion of the two bones. Fusion of the two bones could be observed 1 month after the operation. The area was dominated by newly formed irregular calcification and a loss of original well-defined cortical structure (Figure 12).

Calcification into the defect area could be observed as early as 1 month after the implantation. The calcification began in the defect ends at the same time with the formation of callus. During the 6-month observation period, a slow ingrowth of this calcified tissue into the defect site could be observed. Calcified tissue of about 3–5 mm developing from the ulna into the defect area could be verified with a conventional X-ray 6 months after surgery. Most of these tissues were located in the interior side, in other words in between the area of implanted filler and the radius. There was a gradual increase in density and a slow increase in volume of this calcified tissue during the observation period on the majority of the animal subjects. There was no reunion of the defected ulna after 1 year of healing. By contrast with results obtained in the unmodified polyurethane implanted group, there was calcification in a location away from the radius on the implant. This calcified outgrowth originated in the proximal side of the defect at the ulna, progressing to the distal side, penetrating to about 5–7 mm into the defect area on some samples. There was a significant increase in length of this outgrowth throughout the 1-year observation period, but bridging between the defects was not complete by the end of experimental period (Figure 12).

**Figure 13:**
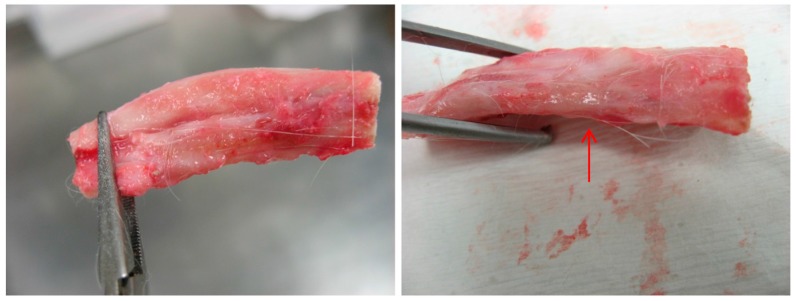
Rabbit fore arm with critical bone defect treated with polyurethane composite filler (PU/PL 2:1) after 6 month’s healing. There is a general restoration of appearance of the original bone. Cartilage-like issue (red arrow) can be found on the implantation.

After scarification, dissection of the animal revealed extensive formation of new tissue around the implantation site (Figure 13). The filler had been completely surrounded by these newly formed tissues, which resemble the anatomy of a normal limb. Despite not being highly calcified, these tissues were tough and had relatively low elasticity. At the proximal side of the implant there existed calcified hard tissue that ran about 1 cm down from the proximal end of the ulna.

**Figure 14:**
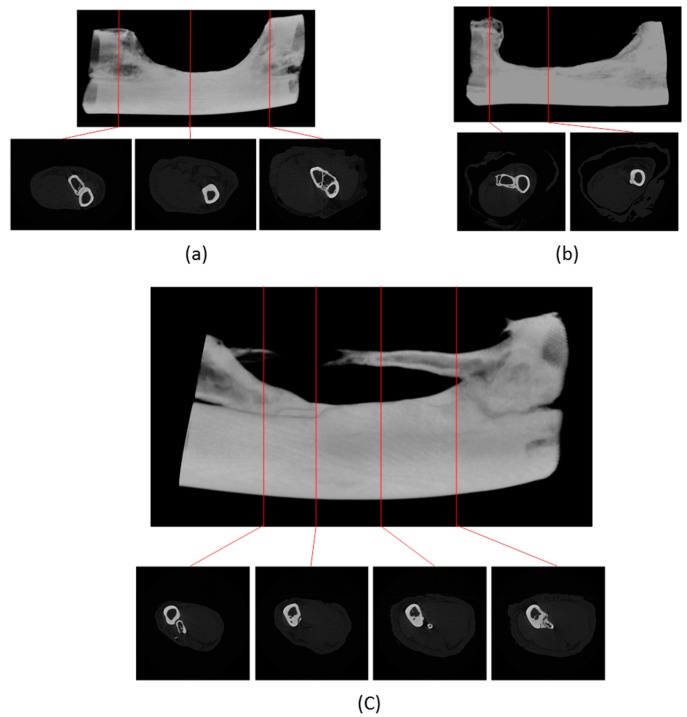
Critical-sized defect after 1 year of healing with differences under micro CT-scanning. (**a**) Control, no implantation; (**b**) polyurethane filler; (**c**) PL/PU 1:2 composite filler.

Micro CT-scanning was performed at 6 months and 1 year after scarification of the animal. The effect of the remodeling process at the defect ends was revealed in the reconstructed images. Thickening of the cortical bone could be observed near the defect area. Next to the site of implantation there was partial loss of cortical structure in the ulna and radius where they were adjacent to each other. The ulna and radius fused into one single bone. Under reconstructed cross sections there was formation of a new calcified trabecular structure within the cortex. New bone formation was more extensive in the proximal end than the distal end of the defect. In a region within 1 cm of the proximal end of defect, newly formed calcified tissue expressed a compact cortex with a narrow central region which resembled the anatomy of compact bone with medullary cavity. After 1 year of healing, the outgrowth reached 1 cm at the exterior side of the defect (Figure 14).

Differences in healing response between the composite and unmodified polyurethane filler are grouped in the following Table 2.

**Figure 15:**
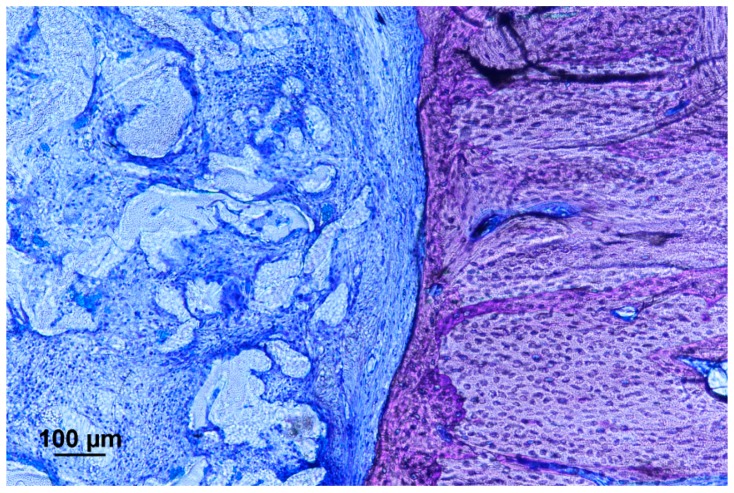
Histological staining in location next to segmental end of the critical-sized defect implanted with a polyurethane scaffold (Giemsa). There is no bone penetration into the scaffold, with the formation of a layer of fibrous tissue around the scaffold.

**Figure 16:**
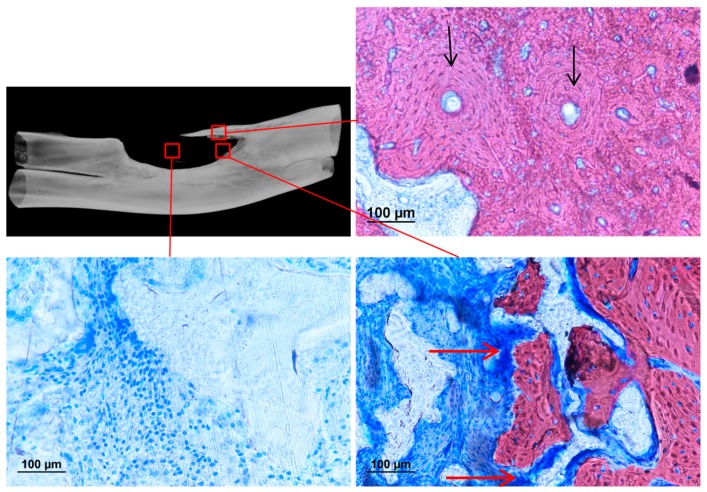
Histological staining of section in a different location inside the defect implanted with polyurethane composite filler (Alizarin red, counter-stained with Toluidine blue, 20×). Osteon can be found in the newly formed bones on the outgrowth (black arrow). Calcification can take place inside the porous system (red arrow).

**Figure 17:**
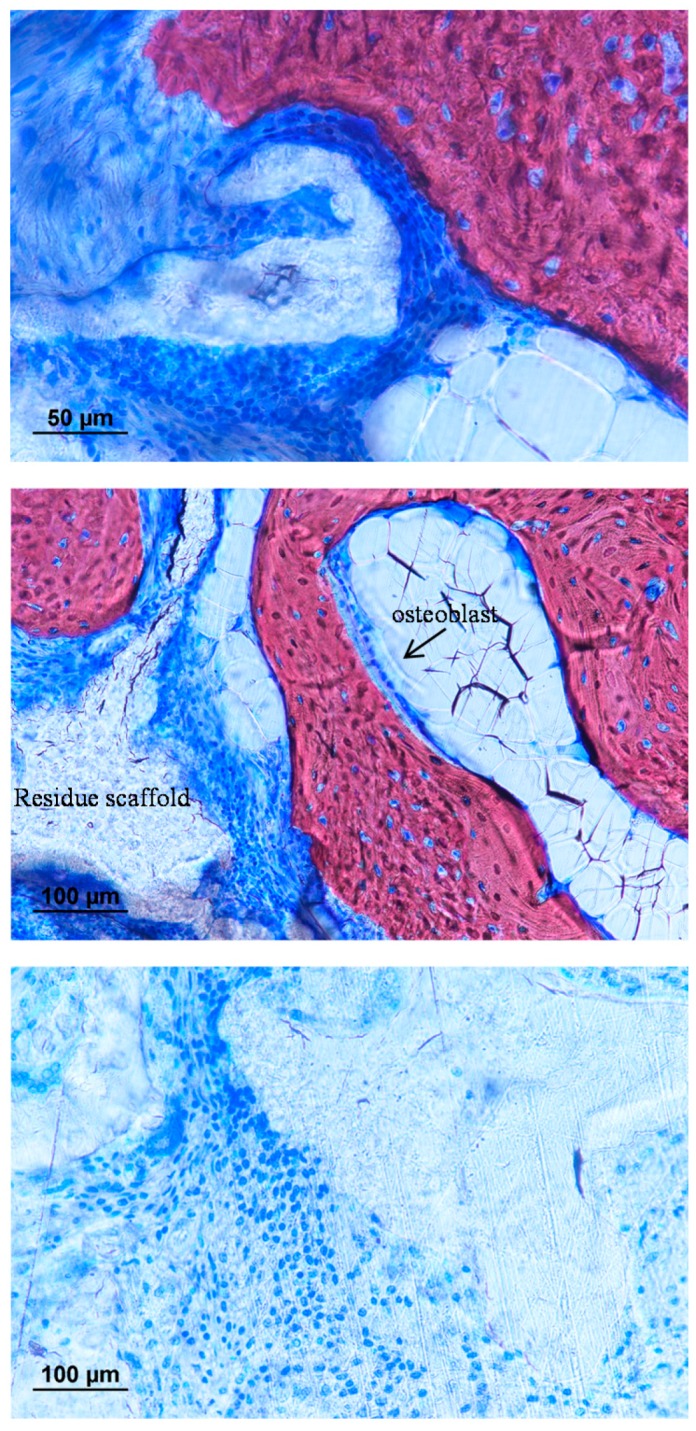
Histological staining in different locations of the critical-sized defect implanted with polyurethane composite filler (Alizarin red, counter stain with Toluidine blue). Osteoblast can be found next to the newly formed bones inside the porous filler.

The presence of calcification was further confirmed under histological staining. Tissue penetration into the scaffold could be observed inside both the pure and composite scaffold, but with a reduction of the fibrous capsule between the bone and composite scaffold interface. (Figure 15). Calcification into the defect area could only be found on the composite group. The bony outgrowth was located on the surface of the implanted filler rather than inside the pores. There was extensive cell penetration into the filler structure but most of the volume remained uncalcified. Fibrous tissue dominated the space inside the porous system. Calcification could be found inside the pores only at the segmental ends of the ulna and was in close contact with the composite filler. (Figure 16 and Figure 17).

## 4. Discussion

Recent developments in tissue regeneration medicine have established various new methods for bone regeneration. Many of these successes relate to the introduction of growth factors, stem cell therapy, as well as a new artificial matrix for tissue proliferation [8,22,23,24,25,26,27,28,29,30]. While natural polymers and ceramics still dominate in the field of bone regeneration, biopolymers such as PLA play an important role thanks to their flexibility and ease of fabrication [8,12,18]. Compared to many other biopolymers, the application of polyurethane in regeneration medicine is relatively rare due to its bio-inert nature and long in vivo degradation time. As a degradable bone filler for orthopedics, polyurethane is usually considered as lacking the mechanical strength for weight-bearing as well as having a low cell adhesion coefficient and long degradation time. However, polyurethane offers unique advantages with its physical properties. Its soft and flexible nature enables it to be fitted into all kinds of defects while at the same time maintaining close contact with surrounding tissue. Tight fitting can also reduce the chances of dislocation during healing. Its high elasticity prevents accumulation of stress inside the defect, minimizing the effect of external stress on the healing process. The toughness and flexibility of the material under long-term implantation suggests high compatibility with healing processes that take a long time. These special advantages raise interest in employing polyurethane as a bone filler for tissue regeneration in critical-sized defects where the weight-bearing issue is less essential. None of the implanted filler in our experiment suffered from dislocation, and it maintained close contact with segmented bone throughout the experimental period. There is no doubt this can help in establishing a more stable interface during the healing process.

To counter the shortcomings of polyurethane, including the bio-inert surface chemistry, long degradation time and low wettability, poly l-lactic acid was incorporated into polyurethane to form a composite porous filler. The resulting filler has different mechanical behavior (reduction in elasticity and strength, increase in resistance to permanent dimension change under stress), but shares the same favorable soft nature of polyurethane. The biological response of the filler could be significantly improved with this composition. Initial cell attachment efficiency as well as cellular metabolism under in vitro culture was significantly improved. There was also an increase in the extracellular matrix deposition and an increase in the expression of osteogenic markers such as alkaline phosphatases activity and calcium deposition. It is suggested that the improvement in osteogenic cellular response was due to the combined effect of surface chemistry modification and an improvement in wettability. Under fluorescent imaging and a scanning electron microscope, we could observe the increase in poly l-lactic acid content encouraging more cells to form lamellipodia with the material surface. This led to the improvement in cell attachment and encouraged seeded cells to express their typical morphology, which is beneficial for cell metabolism.

When compared to unmodified polyurethane filler, there was an increase in the degradation rate, which is more compatible with the regeneration rate of bone. The most significant finding in the animal study was that polyurethane composite filler can promote the formation of calcification bridging across critical-sized defects. With pure polyurethane filler, calcification and bone regeneration is missing due to the bio-inertness of polyurethane, which has already been described in related studies [51]. This setback can be significantly overcome by incorporating the poly l-lactic acid. After modification, calcified tissue can propagate on the new material with osteoblasts penetrating into the porous system. Intra-matrix calcification could also be confirmed under micro CT-scanning and histological staining, which suggested the filler can encourage a certain degree of calcification. Formation of these calcifications around the filler indicated an improvement in osteoconductivity. As with the result of in vitro tests, this improvement is believed to be due to the improvement in the biological response of the modified polyurethane composite.

It is not disputed that the result of the in vivo experiment presented here cannot achieve the same degree of recovery as that found in some of the published literature on similar animal models [25]. The major reason for this is that no osteogenic growth factors and stem cells were introduced in this study, which significantly reduced the amount of calcification and the healing rate. Various literature supports the idea that the introduction of rhBMP can promote calcification and bone regeneration in large defects [25,66,67,68,69,70,71]. The introduction of mesenchymal stem cells can also significantly improve bone formation within these defects [69]. Despite a lesser degree of calcification in our result when compared with biological incorporated systems, dissection confirmed that there is guided connective tissue formation across the defect gap that resembled the anatomy of natural bone. The presented filler is comparable, or can offer an advantage in anatomical reconstruction as well as calcified tissue formation when compared to some bone graft substituting biopolymers [72,73,74].

In overall terms, the biological response of polyurethane can be effectively improved by incorporating poly l-lactic acid, while at the same time retaining the feasible physical properties of polyurethane. This produces a flexible soft scaffold capable of fitting in most bone defects with a low chance of dislocation and acceptable osteoconductivity. Investigation on animal models indicated that the composite filler can facilitate calcification inside artificially created critical-sized defects even without the introduction of cells and growth factors. The results demonstrate the potential of this polyurethane composite as a filler material for healing facilitation across critical-sized bone defects.

## Figures and Tables

**Table 1:** Weight ratio of different polyurethane composites used in the investigation.

Code Name	Weight Ratio, Polyurethane (PU):Poly l-Lactic Acid (PL)
PU	1:0
PL/PU 1:4	1:0.25
PL/PU 1:2	1:0.5
PL/PU 3:4	1:0.75
PL/PU 1:1	1:1

**Table 2:** Summary of healing response of composite and un-modified polyurethane filler in critical-sized bone defect.

Healing Response	PL/PU Composite Filler	Unmodified Polyurethane Filler
Dislocation	-	-
Necrosis	-	-
Fibrous tissue inside pore system	+	+
Cell ingrowth	+	+
Calcification inside the porous system	+	-
Bone formation across the defect	+	-

## References

[1] Bosch C., Melsen B., Vargervik K. (1998). Importance of the critical-size bone defect in testing bone-regenerating materials. J. Craniofac. Surg..

[2] Pape H.C., Pufe T. (2010). Bone defects and nonunions-What role does vascularity play in filling the gap?. Inj. Int. J. Care Inj..

[3] Wiese A., Pape H.C. (2010). Bone Defects Caused by High-energy Injuries, Bone Loss, Infected Nonunions, and Nonunions. Orthop. Clin. N. Am..

[4] Giannoudis P.V. (2016). Treatment of bone defects: Bone transport or the induced membrane technique?. Inj. Int. J. Care Inj..

[5] Dimitriou R., Mataliotakis G.I., Angoules A.G., Kanakaris N.K., Giannoudis P.V. (2011). Complications following autologous bone graft harvesting from the iliac crest and using the RIA: A systematic review. Inj. Int. J. Care Inj..

[6] Arrington E.D., Smith W.J., Chambers H.G., Bucknell A.L., Davino N.A. (1996). Complications of iliac crest bone graft harvesting. Clin. Orthop. Relat. Res..

[7] Sen M.K., Miclau T. (2007). Autologous iliac crest bone graft: Should it still be the gold standard for treating nonunions?. Inj. Int. J. Care Inj..

[8] Nandi S.K., Roy S., Mukherjee P., Kundu B., De D.K., Basu D. (2010). Orthopaedic applications of bone graft & graft substitutes: A review. Indian J. Med. Res..

[9] Zimmermann G., Moghaddam A. (2011). Allograft bone matrix versus synthetic bone graft substitutes. Injury.

[10] Viateau V., Guillemin G., Bousson V., Oudina K., Hannouche D., Sedel L. (2007). Long-bone critical-size defects treated with tissue-engineered grafts: A study on sheep. J. Orthop. Res..

[11] Zhang Z.Y., Teoh S.H., Chong M.S.K., Lee E.S.M., Tan L.G., Mattar C.N., Fisk N.M., Choolani M., Chan J. (2010). Neo-vascularization and bone formation mediated by fetal mesenchymal stem cell tissue-engineered bone grafts in critical-size femoral defects. Biomaterials.

[12] Burg K.J.L., Porter S., Kellam J.F. (2000). Biomaterial developments for bone tissue engineering. Biomaterials.

[13] Clokie C.M.L., Moghadam H., Jackson M.T., Sandor G.K.B. (2002). Closure of critical sized defects with allogenic and alloplastic bone substitutes. J. Craniofac. Surg..

[14] Furlaneto F.A.C., Nagata M.J.H., Fucini S.E., Deliberador T.M., Okamoto T., Messora M.R. (2007). Bone healing in critical-size defects treated with bioactive glass/calcium sulfate: A histologic and histometric study in rat calvaria. Clin. Oral Implant. Res..

[15] Ye J.H., Xu Y.J., Gao J., Yan S.G., Zhao J., TU Q.S., Tu J., Zhang J., Duan X.J., Sommer C.A. (2011). Critical-size calvarial bone defects healing in a mouse model with silk scaffolds and SATB2-modified iPSCs. Biomaterials.

[16] Schubert T., Lafont S., Beaurin G., Grisay G., Behets C., Gianello P., Dufrane D. (2013). Critical size bone defect reconstruction by an autologous 3D osteogenic-like tissue derived from differentiated adipose MSCs. Biomaterials.

[17] Intini G., Andreana S., Buhite R.J., Bobek L.A. (2008). A comparative analysis of bone formation induced by human demineralized freeze-dried bone and enamel matrix derivative in rat calvaria critical-size bone defects. J. Periodontol..

[18] Giannoudis P.V., Dinopoulos H., Tsiridis E. (2005). Bone substitutes: An update. Inj. Int. J. Care Inj..

[19] Yuan H.P., Fernandes H., Habibovic P., de Boer J., Barradas A.M.C., de Ruiter A., Walsh W.R., van Blitterswijk C.A., de Bruijn J.D. (2010). Osteoinductive ceramics as a synthetic alternative to autologous bone grafting. Proc. Natl. Acad. Sci. USA.

[20] Greenwald A.S., Boden S.D., Goldberg V.M., Khan Y., Laurencin C.T., Rosier R.N. (2001). Bone-graft substitutes: Facts, fictions, and applications. J. Bone Jt. Surg. Am..

[21] Damien C.J., Parsons J.R. (1991). Bone graft and bone graft substitutes: A review of current technology and applications. J. Appl. Biomater..

[22] Dilogo I.H., Primaputra M.R.A., Pawitan J.A., Liem I.K. (2017). Modified Masquelet technique using allogeneic umbilical cord-derived mesenchymal stem cells for infected non-union femoral shaft fracture with a 12 cm bone defect: A case report. Int. J. Surg. Case Rep..

[23] Henriques Lourenco A., Neves N., Ribeiro-Machado C., Sousa S.R., Lamghari M., Barrias C.C., Cabral A.T., Barbosa M.A., Ribeiro C.C. (2017). Injectable hybrid system for strontium local delivery promotes bone regeneration in a rat critical-sized defect model. Sci. Rep..

[24] Yoon E., Dhar S., Chun D.E., Gharibjanian N.A., Evans G.R. (2007). In vivo osteogenic potential of human adipose-derived stem cells/poly lactide-co-glycolic acid constructs for bone regeneration in a rat critical-sized calvarial defect model. Tissue Eng..

[25] Liu T., Wu G., Gu Z., Wismeijer D., Liu Y. (2014). A critical-sized bone defect. Bone.

[26] Sathy B.N., Watson M., Kinard L.A., Spicer P.P., Dahlin R.L., Mikos A.G., Nair S. (2015). Bone Tissue Engineering with Multilayered Scaffolds-Part II: Combining Vascularization with Bone Formation in Critical-Sized Bone Defect. Tissue Eng. Part A.

[27] Van de Watering F.C., van den Beucken J.J., Walboomers X.F., Jansen J.A. (2012). Calcium phosphate/poly(d,l-lactic-co-glycolic acid) composite bone substitute materials: Evaluation of temporal degradation and bone ingrowth in a rat critical-sized cranial defect. Clin. Oral Implant. Res..

[28] Auregan J.C., Begue T. (2014). Induced membrane for treatment of critical sized bone defect: A review of experimental and clinical experiences. Int. Orthop..

[29] Jannetty J., Kolb E., Boxberger J., Deslauriers R., Ganey T. (2010). Guiding bone formation in a critical-sized defect and assessments. J. Craniofac. Surg..

[30] Skovrlj B., Guzman J.Z., Maaieh M.A., Cho S.K., Iatridis J.C., Qureshi S.A. (2014). Cellular bone matrices: Viable stem cell-containing bone graft substitutes. Spine J..

[31] Hollinger J.O., Brekke J., Gruskin E., Lee D. (1996). Role of bone substitutes. Clin. Orthop. Relat. Res..

[32] Kurien T., Pearson R.G., Scammell B.E. (2013). Bone graft substitutes currently available in orthopaedic practice: The evidence for their use. Bone Jt. J..

[33] Finkemeier C.G. (2002). Bone-grafting and bone-graft substitutes. J. Bone Jt. Surg. Am..

[34] Gupta A., Kukkar N., Sharif K., Main B.J., Albers C.E., El-Amin Iii S.F. (2015). Bone graft substitutes for spine fusion: A brief review. World J. Orthop..

[35] Coombes A.G., Meikle M.C. (1994). Resorbable synthetic polymers as replacements for bone graft. Clin. Mater..

[36] Laurencin C., Khan Y., El-Amin S.F. (2006). Bone graft substitutes. Expert Rev. Med. Devices.

[37] Hollinger J.O., Battistone G.C. (1986). Biodegradable bone repair materials. Synthetic polymers and ceramics. Clin. Orthop. Relat. Res..

[38] Eppley B.L., Sadove A.M. (1995). A comparison of resorbable and metallic fixation in healing of calvarial bone grafts. Plast. Reconstr. Surg..

[39] Kim S.S., Park M.S., Jeon O., Choi C.Y., Kim B.S. (2006). Poly(lactide-co-glycolide)/hydroxyapatite composite scaffolds for bone tissue engineering. Biomaterials.

[40] Miki T., Harada K., Imai Y., Enomoto S. (1994). Effect of freeze-dried poly-l-lactic acid discs mixed with bone morphogenetic protein on the healing of rat skull defects. J. Oral Maxillofac. Surg..

[41] Chen J., Chu B., Hsiao B.S. (2006). Mineralization of hydroxyapatite in electrospun nanofibrous poly(l-lactic acid) scaffolds. J. Biomed. Mater. Res. A.

[42] Hasegawa Y., Ohgushi H., Ishimura M., Habata T., Tamai S., Tomita N., Ikada Y. (1999). Marrow cell culture on poly-l-lactic acid fabrics. Clin. Orthop. Relat. Res..

[43] Van Dijk M., Smit M.T.H., Burger E.H., Wuisman P.I. (2002). Bioabsorbable poly-l-lactic acid cages for lumbar interbody fusion: Three-year follow-up radiographic, histologic, and histomorphometric analysis in goats. Spine (Phila Pa 1976).

[44] Mackay T.G., Wheatley D.J., Bernacca G.M., Fisher A.C., Hindle C.S. (1996). New polyurethane heart valve prosthesis: Design, manufacture and evaluation. Biomaterials.

[45] Pavlova M., Draganova M. (1994). Hydrolytic stability of polyurethane medical adhesive dressings. Biomaterials.

[46] Khil M.S., Cha D.I., Kim H.Y., Kim I.S., Bhattarai N. (2003). Electrospun nanofibrous polyurethane membrane as wound dressing. J. Biomed. Mater. Res. Part B Appl. Biomater..

[47] Coury A.J., Slaikeu P.C., Cahalan P.T., Stokes K.B., Hobot C.M. (1988). Factors and interactions affecting the performance of polyurethane elastomers in medical devices. J. Biomater. Appl..

[48] Gogolewski S. (1987). Implantable Segmented Polyurethanes—Controversies and Uncertainties. Life Support Syst..

[49] Gorna K., Gogolewski S. (2003). Preparation, degradation, and calcification of biodegradable polyurethane foams for bone graft substitutes. J. Biomed. Mater. Res. A.

[50] Gorna K., Gogolewski S. (2006). Biodegradable porous polyurethane scaffolds for tissue repair and regeneration. J. Biomed. Mater. Rese. Part A.

[51] Nielsen F.F., Karring T., Gogolewski S. (1992). Biodegradable Guide for Bone Regeneration—Polyurethane Membranes Tested in Rabbit Radius Defects. Acta Orthop. Scand..

[52] Roohpour N., Wasikiewicz J.M., Paul D., Vadgama P., Rehman I.U. (2009). Synthesis and characterisation of enhanced barrier polyurethane for encapsulation of implantable medical devices. J. Mater. Sci. Mater. Med..

[53] Kutting M., Roggenkamp J., Urban U., Schmitz-Rode T., Steinseifer U. (2011). Polyurethane heart valves: Past, present and future. Expert Rev. Med. Devices.

[54] Cruz C. (1988). The peritoneoscopic implantation of a polyurethane percutaneous access device for peritoneal dialysis. Preliminary experience. ASAIO Trans..

[55] Peng C.W., Tan S.G. (2003). Polyurethane grafts: A viable alternative for dialysis arteriovenous access?. Asian Cardiovasc. Thorac. Ann..

[56] Coury A.J., Stokes K.B., Cahalan P.T., Slaikeu P.C. (1987). Biostability considerations for implantable polyurethanes. Life Support Syst..

[57] Howard G.T. (2002). Biodegradation of polyurethane: A review. Int. Biodeterior. Biodegrad..

[58] Bacakova L., Filova E., Rypacek F., Svorcik V., Stary V. (2004). Cell adhesion on artificial materials for tissue engineering. Physiol. Res..

[59] Gogolewski S., Gorna K., Turner A.S. (2006). Regeneration of bicortical defects in the iliac crest of estrogen-deficient sheep, using new biodegradable polyurethane bone graft substitutes. J. Biomed. Mater. Res. Part A.

[60] Hsu S.H., Chen W.C. (2000). Improved cell adhesion by plasma-induced grafting of l-lactide onto polyurethane surface. Biomaterials.

[61] Gorna K., Gogolewski S. (2003). Molecular stability, mechanical properties, surface characteristics and sterility of biodegradable polyurethanes treated with low-temperature plasma. Polym. Degrad. Stab..

[62] Mohan B.G., Shenoy S.J., Babu S.S., Varma H.K., John A. (2013). Strontium calcium phosphate for the repair of leporine (*Oryctolagus cuniculus*) ulna segmental defect. J. Biomed. Mater. Res. Part A.

[63] Roohani-Esfahani S.I., Dunstan C.R., Davies B., Pearce S., Williams R., Zreiqat H. (2012). Repairing a critical-sized bone defect with highly porous modified and unmodified baghdadite scaffolds. Acta Biomater..

[64] Hafezi F., Hosseinnejad F., Fooladi A.A.I., Mafi S.M., Amiri A., Nourani M.R. (2012). Transplantation of nano-bioglass/gelatin scaffold in a non-autogenous setting for bone regeneration in a rabbit ulna. J. Mater. Sci.-Mater. Med..

[65] Bodde E.W.H., Spauwen P.H.M., Mikos A.G., Jansen J.A. (2008). Closing capacity of segmental radius defects in rabbits. J. Biomed. Mater. Res. Part A.

[66] Liu T., Zheng Y., Wu G., Wismeijer D., Pathak J.L., Liu Y. (2017). BMP2-coprecipitated calcium phosphate granules enhance osteoinductivity of deproteinized bovine bone, and bone formation during critical-sized bone defect healing. Sci. Rep..

[67] Muller C.W., Hildebrandt K., Gerich T., Krettek C., van Griensven M., Balmayor E.R. (2017). BMP-2-transduced human bone marrow stem cells enhance neo-bone formation in a rat critical-sized femur defect. J. Tissue Eng. Regen. Med..

[68] Bougioukli S., Jain A., Sugiyama O., Tinsley B.A., Tang A.H., Tan M.H., Adams D.J., Kostenuik P.J., Lieberman J.R. (2016). Combination therapy with BMP-2 and a systemic RANKL inhibitor enhances bone healing in a mouse critical-sized femoral defect. Bone.

[69] Gao X., Usas A., Lu A., Tang Y., Wang B., Chen C.W., Li H., Tebbets J.C., Cummins J.H., Huard J. (2013). BMP2 is superior to BMP4 for promoting human muscle-derived stem cell-mediated bone regeneration in a critical-sized calvarial defect model. Cell Transpl..

[70] Cicciu M., Herford A.S., Juodzbalys G., Stoffella E. (2012). Recombinant human bone morphogenetic protein type 2 application for a possible treatment of bisphosphonates-related osteonecrosis of the jaw. J. Craniofac. Surg..

[71] Herford A.S., Cicciu M. (2010). Recombinant human bone morphogenetic protein type 2 jaw reconstruction in patients affected by giant cell tumor. J. Craniofac. Surg..

[72] El Backly R.M., Chiapale D., Muraglia A., Tromba G., Ottonello C., Santolini F., Cancedda R., Mastrogiacomo M. (2014). A modified rabbit ulna defect model for evaluating periosteal substitutes in bone engineering: A pilot study. Front. Bioeng. Biotechnol..

[73] Lee J.Y., Son S.J., Son J.S., Kang S.S., Choi S.H. (2016). Bone-Healing Capacity of PCL/PLGA/Duck Beak Scaffold in Critical Bone Defects in a Rabbit Model. Biomed. Res. Int..

[74] Chen S.H., Lau P.Y., Lei M., Peng J., Tang T., Wang X.H., Qin L., Kumta S.M. (2017). Segmental composite porous scaffolds with either osteogenesis or anti-bone resorption properties tested in a rabbit ulna defect model. J. Tissue Eng. Regen. Med..

